# Sampling and composition of airborne particulate matter (PM_10_) from two locations of Mexico City

**DOI:** 10.1016/j.dib.2015.06.017

**Published:** 2015-07-02

**Authors:** Yolanda I. Chirino, Yesennia Sánchez-Pérez, Álvaro Román Osornio-Vargas, Irma Rosas, Claudia María García-Cuellar

**Affiliations:** a Unidad de Biomedicina, Facultad de Estudios Superiores Iztacala, Universidad Nacional Autónoma de México, Los Reyes Iztacala, CP 54090 Tlalnepantla, Estado de México, México; b Instituto Nacional de Cancerología (INCan), Subdirección de Investigación Básica, San Fernando No. 22, Tlalpan, 14080 México, D.F., México; c Department of Pediatrics, University of Alberta, 3-591 ECHA, 11405 87th Avenue, Edmonton, Canada T6G 1C9; d Centro de Ciencias de la Atmósfera, Universidad Nacional Autónoma de México (UNAM), Circuito Exterior s/n. Ciudad Universitaria, Del. Coyoacán, C.P. 04510, Mexico, D.F., Mexico

## Abstract

The PM_10_ airborne particulate matter with an aerodynamic diameter ≤10 µm is considered as a risk factor of various adverse health outcomes, including lung cancer. Here we described the sampling and composition of PM_10_ collected from an industrial zone (IZ), and a commercial zone (CZ) of Mexico City. The PM_10_ was collected with a high-volume sampler in the above mentioned locations and both types of PM_10_ sampled were characterized by the content of polycyclic aromatic hydrocarbons (PAHs), metals, and endotoxin. The endotoxin PM_10_ content from IZ and CZ displayed 138.4 UE/mg and 170.4 UE/mg of PM_10,_ respectively.

Specifications table

**Table:** 

Subject area	Biology
More specific subject area	PM_10_ sampling and description of polycyclic aromatic hydrocarbons, metals and endotoxin content.
Type of data	Text file and figures.
How data were acquired	Data were acquired used through a kinetic assay of Limulus Amebocyte lysate (BioWhittaker, Walkersville, MD, USA) using *Escherichia coli* endotoxin as standard. The optical density of each well was recorded at a wavelength of 405 nm every 150 s. The microplate reader was controlled and data were recorded by a Gateway 450 PC-XT computer.
Data format	Analyzed.
Data source location	The samples were collected from Mexico City.
Data accessibility	The data are within this paper.

Value of the data

• Air quality can be evaluated partially by the content of particulate matter with an aerodynamic diameter ≤10 µm (PM_10_).
• Specific PM_10_ components could be responsible for effects on human health.
• PM_10_ sampled reveals the presence of harmful components such as polycyclic aromatic hydrocarbons (PAHs) and oxidant metals.

Data

Here we describe the sampling and composition of PM_10_ collected from an industrial zone (IZ), and a commercial zone (CZ) of Mexico City.

## 1 Experimental design, materials and methods

### 1.1 PM_10_ sampling

Particulate matter with an aerodynamic size of 10 μm (PM_10_) was collected using a high-volume sampler [1] from an Industrial zone (IZ) located in the northern part of Mexico City, where several industries are located. This area, which includes some neighboring municipalities of Xalostoc in the State of Mexico, harbors a wide variety of industries related to steel, grinding minerals, plastic manufacturing, industrial soap production, concrete, and cleaning products.

The other selected area for collecting particles is a commercial zone (CZ) located in the neighborhood called *La Merced*, which is a traditional food market area found closely to the Mexico City Historic downtown. Every day, several trucks with diesel and gasoline engines deliver food products in this area. Also, there is an important bus terminal station (Terminal de Autobuses de Pasajeros de Oriente) in this area. In addition, there is a heavy private and public transportation based on diesel or gasoline engines constituting an additional source of pollution in this area.

**Fig. 1:**
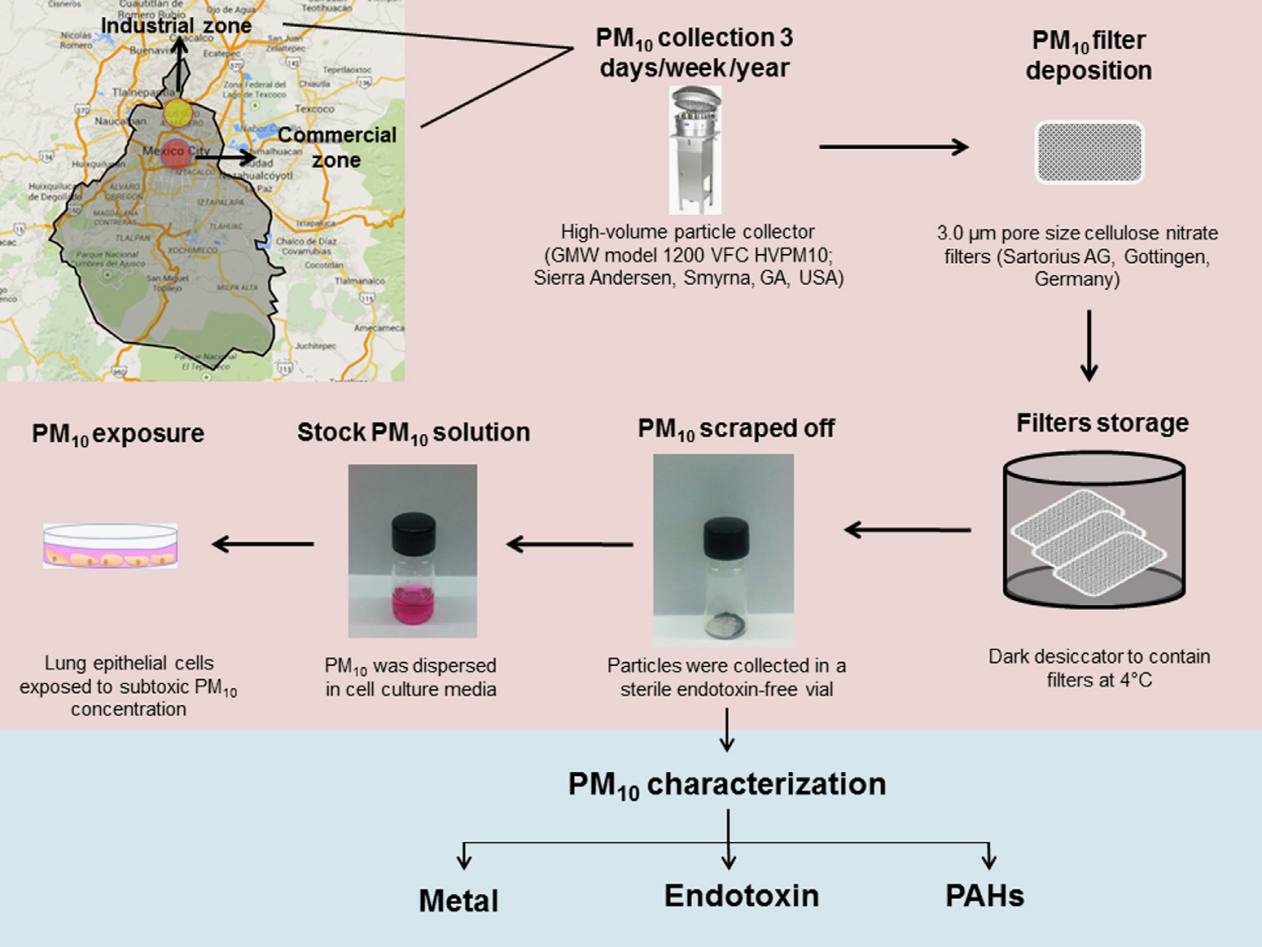
Particulate matter with aerodynamic diameter of 10 μm (PM_10_) was sampled from an industrial zone (yellow circle) and commercial zone (red circle) in Mexico City (shadowy outline in the map). Particle collector was used to sample PM_10_ from October 2004 to May 2005 in cellulose nitrate filters, which were kept in dark desiccators at 4 °C. Then, PM_10_ was scrapped off from filters and maintained in sterile vials until usage. Before experiments, PM_10_ contained in vials were sterilized and resuspended in cell culture medium for in vitro experiments or used for characterization in order to determine PM components.

Sample collection of PM_10_ was performed using one cellulose nitrate filter per day, excluding the rainy season (June–September). PM_10_ from the whole period (October–May) was recovered from filters and a PM_10_-year pool was stored in free-endotoxin sterile vials at 4 °C until usage for experimentation and physicochemical characterization (see Fig. 1).

### 1.2 PM_10_ composition

**Fig. 2:**
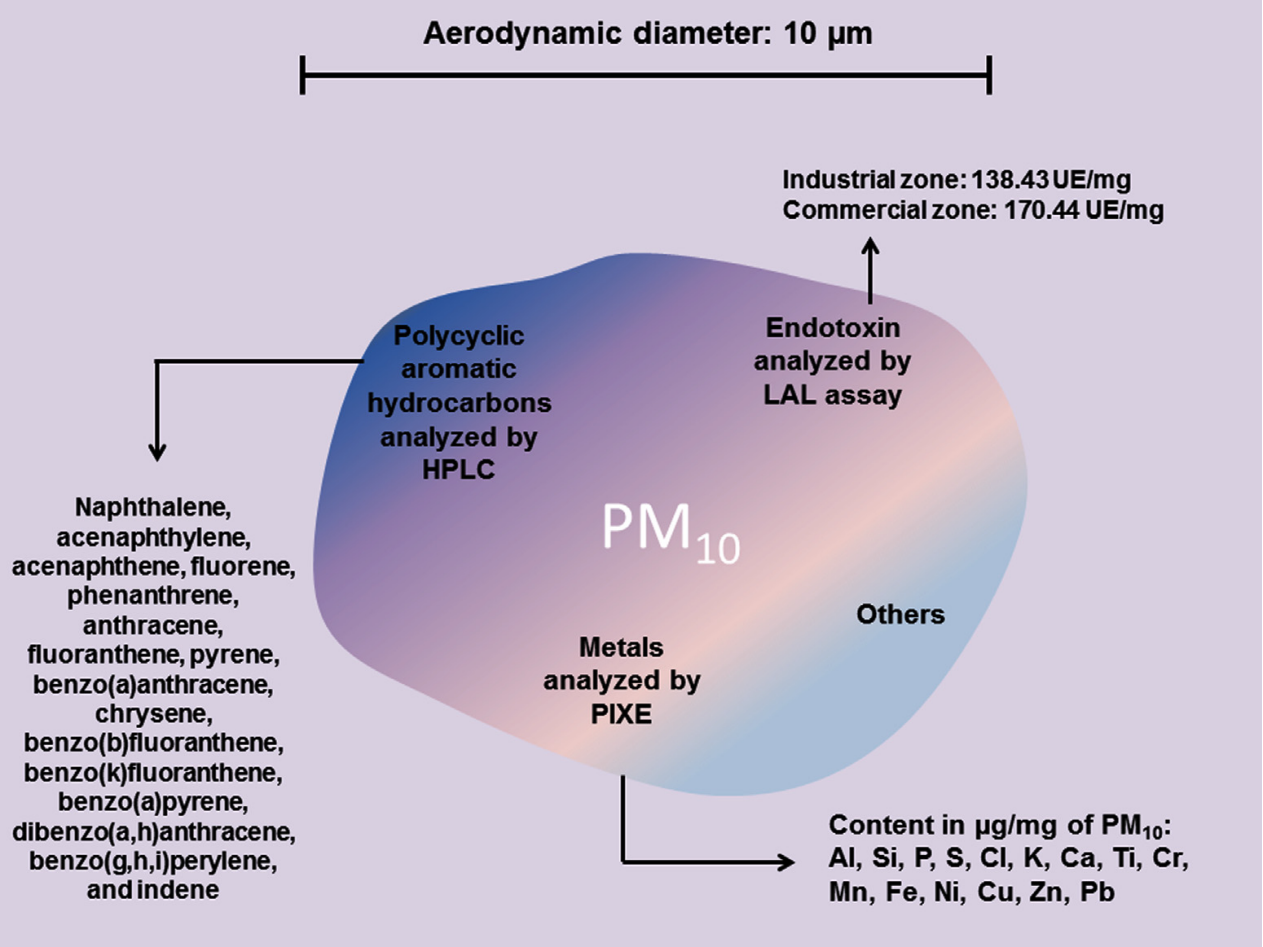
The composition of particulate matter with aerodynamic diameter of 10 μm (PM_10_). PM_10_ is a complex mixture of polycyclic aromatic hydrocarbons (PAHs), metals and endotoxin, among others. PAHs were analyzed by high-pressure liquid chromatography (HPLC); metals by particle-induced X-ray emission (PIXE) analysis, and endotoxin content was analyzed by limulus amebocyte lysate (LAL) assay.

The characterization of collected particles was initially performed to identify the main components of PM_10_, which are related to polycyclic aromatic hydrocarbons (PAHs), metals and endotoxin. Then, using dichloromethane extraction followed by high-pressure liquid chromatography (HPLC; Agilent HP, 1100 series) PAHs content was determined [2]. Elemental metal analysis to the pool of PM_10_ [3] was performed by particle-induced X-ray emission (PIXE) using a proton beam produced by a 9SDH-2 Pelletron accelerator. Endotoxin content was measured through a kinetic assay of Limulus Amebocyte lysate (LAL) assay according to the manufacturer׳s specifications (BioWhittaker, Walkersville, MD, USA) using *Escherichia coli* endotoxin as standard [4]. We have reported some of the most representative compounds of PM_10_, which includes PAHs, endotoxin and aluminum (Al), silicon (Si), phosphorus (P), sulfur (S), chlorine (Cl), potassium (K), calcium (Ca), titanium (Ti), chrome (Cr), manganese (Mn), iron (Fe), nickel (Ni), copper (Cu), zinc (Zn) and lead (Pb). Endotoxin results showed that PM_10_ collected from both the industrial zone and the commercial zone displayed 138.4 UE/mg and 170.4 UE/mg of PM_10,_ respectively (Fig. 2).

Importance of PM_10_ composition lies on the possibility to explain that biological effects associated to inhalatory exposure are different among polluted cities. Some of these effects are specially alarming because can be related to the acquisition of new characteristics such as invasiveness by targeted PM_10_ cells, such as lung epithelial cells and PM_10_ derived from one location can even be more harmful that other [5].
